# Targeting uroporphyrinogen decarboxylase for head and neck cancer treatment

**DOI:** 10.1186/1753-6561-7-S2-P19

**Published:** 2013-04-04

**Authors:** Emma Ito, Shijun Yue, Eduardo H Moriyama, Angela B Hui, Inki Kim, Wei Shi, Nehad M Alajez, Nirmal Bhogal, GuoHua Li, Alessandro Datti, Aaron D Schimmer, Brian C Wilson, Peter P Liu, Daniel Durocher, Benjamin G Neel, Brian O’Sullivan, Bernard Cummings, Rob Bristow, Jeff Wrana, Fei-Fei Liu

**Affiliations:** 1 Department of Medical Biophysics, University of Toronto, Toronto, Canada; 2 Ontario Cancer Institute, Campbell Family Cancer Research Institute, University Health Network, Toronto, Canada; 3 Toronto General Research Institute, University Health Network, Toronto, Canada; 4 Samuel Lunenfeld Research Institute, Mount Sinai Hospital, Toronto, Canada; 5 Department of Experimental Medicine and Biochemical Sciences, University of Perugia, Perugia, Italy; 6 Department of Radiation Oncology, Princess Margaret Hospital, University Health Network, Toronto, Canada; 7 Department of Radiation Oncology, University of Toronto, Toronto, Canada

## Background

Head and neck cancer (HNC) is the 8^th^ most common malignancy worldwide. Despite advances in therapeutic options over the last few decades, treatment toxicities and overall clinical outcomes have remained disappointing, underscoring a need to develop novel therapeutic approaches, particularly those that enhance tumor cell death, while minimizing damage to the surrounding normal tissues.

## Materials and methods

An RNA interference (RNAi)-based high-throughput screen (HTS) was performed for the large-scale identification of novel genes that will selectively sensitize HNC cells to ionizing radiation. The Dharmacon Protein Kinase and Druggable Genome siRNA Libraries were screened using FaDu cells (human hypopharyngeal squamous cell cancer). Radiosensitizing targets were subjected to *in vitro* and *in vivo* characterizations.

## Results

Sixty-seven target sequences with potential radiosensitizing effects were identified. Targets reducing the surviving fraction by >50% at 2 Gy relative to their un-irradiated counterparts were selected for further evaluation. A key regulator of heme biosynthesis, uroporphyrinogen decarboxylase (UROD), was thereby identified as a novel tumor-selective radiosensitizing target, demonstrating both *in vitro* and *in vivo* efficacy. Radiosensitization appeared to be mediated *via* enhancement of tumor oxidative stress from perturbation of iron homeostasis and increased free radical production. UROD was significantly over-expressed in HNC patient biopsies, wherein lower pre-radiation mRNA levels correlated with improved survival, suggesting UROD could potentially predict radiation response. UROD down-regulation also radiosensitized several different human cancer models, while sparing normal cells.

## Conclusions

An RNAi-based radiosensitizer HTS has revealed UROD as a potent tumor-selective sensitizer for radiation, with potential relevance to many human malignancies.

## Financial support

Canadian Institutes of Health Research (CIHR; grant 69023); Elia Chair in Head and Neck Cancer Research; philanthropic support from Wharton Family, J. Finley, and G. Tozer; Campbell Family Institute for Cancer Research; Ministry of Health and Long-Term Planning; CIHR Resource Maintenance grant (PRG-82679).

